# Effects of starters with different NDF/starch ratio on rumen fermentation parameters and rumen microorganisms in lambs

**DOI:** 10.3389/fvets.2023.1064774

**Published:** 2023-01-26

**Authors:** Haibi Zhao, Feng Lv, Guohua Liu, Xin Pang, Xiaoxia Han, Xiaojuan Wang

**Affiliations:** College of Animal Science and Technology, Gansu Agricultural University, Lanzhou, China

**Keywords:** starter, lambs, rumen microbes, metagenomics, NDF/starch

## Abstract

Starch and NDF are the main components in the diets of ruminants worldwide and are the main energy source for rumen microorganisms and hosts. The purpose of this study was to investigate the effects of different NDF/starch ratios on rumen fermentation parameters, rumen development and rumen microbes in lambs and to predict the function of rumen microbes by metagenomic techniques. In this study, 30 lambs with birth weights of (3.0 ± 0.5) kg were selected. The lambs of Hu sheep were randomly divided into two groups, fed starter with an NDF/starch ratio of 0.5 (group A) or 1.0 (group C). Samples of the rumen tissue and contents were collected after slaughter. The results showed that the ADG and ADFI of group A were significantly higher than those of group C (*P* < 0.05), but there was no significant difference in the FCR (*P* > 0.05). Therefore, from the perspective of feed-related economic benefits, group C showed greater economic value; the A/P of group C was significantly lower than that of group A (0.05 < *P* < 0.1), and the TVFA showed no significant difference (*P* > 0.05); The lengths of the rumen papillae of group C was significantly higher than that of group A (0.05 < *P* < 0.1). There was no significant difference in the abundance of the top 10 species at the phylum level and genus level (*P* > 0.05). CAZymes gene enrichment was observed in the rumen microbial community of lambs in group C (*P* < 0.05). In conclusion, group C, fed with starter with a higher NDF/starch ratio, had a higher feeding value. This study provides comprehensive insights into the composition of NDF and starch in lamb starter.

## Introduction

The Hu sheep is an extremely inbred species and the most popular species in China due to its excellent prolificacy (3–4 lambs per parturition), rapid growth rate, and adaptation to poor-quality feeds. Young ruminants are born with underdeveloped rumens and are considered functional monogastric animals before weaning, but rumen development in young ruminants plays a crucial role in animal health and nutrition (1). Colonization of the gastrointestinal tract of young ruminants begins at birth and continues through successive colonization and floristic changes until the microbiota reaches a stable state later in life, and changes in early succession of primary colonizers affect the composition and function of the mature animal community (2). The rumen epithelium is a key barrier between the host and the rumen environment. It can provide energy for ruminants by using the metabolites of rumen microorganisms, such as acetic acid, propionic acid and butyric acid, which are produced by fermentation of microorganisms (3). The rumen epithelium influences the use of nutrients throughout the body by absorbing VFAs (3, 4). VFAs can provide energy for the host and stimulate the development of the rumen epithelium. Early dietary intervention is crucial to the formation of the microflora and has a great impact on improving the performance of young ruminants. The early use of starter is very important for rumen functional development and optimal growth of lambs. Determination of the appropriate nutrient level and preparation of the appropriate feed for rumen development are beneficial for rumen development and for complete rumen microbial function in lambs (5). Neutral detergent fiber (NDF), as the main component of structural carbohydrates in forage, is a vital factor impacting the performance of young ruminants (6). Studies have shown that rumen fermentation levels and the promotion of rumen development can be positively regulated by adjusting dietary NDF in the early life stages of calves and lambs, including adjustments in the levels and sources of NDF (7). The main components of starch are non-structural carbohydrates, which can be rapidly degraded in the rumen to provide energy for lambs and rumen microbes and produce volatile fatty acids (VFAs) to stimulate the development of rumen papilla (7). In ruminants, starch is fermented to VFAs in the rumen, which may provide energy for epithelia and the host animal (8). Early use of starter is very important for the development of rumen function and the optimal growth of lambs. Determining the appropriate feed level and modulating the appropriate carbohydrate level for rumen development are beneficial to the development of lambs and promotion of rumen microbial function (5). Diet is one of the main factors leading to changes in the rumen microbial community (9). Considerable research has focused on cataloging the adult rumen microbiome and its relationship to complex diets (9, 10), and studies on the lamb rumen microbiota have been restricted to 16 S rRNA amplicon sequencing and/or different diets and hosts (11, 12). Although emerging studies have been able to use metagenomics to identify the rumen microbiome at the structural and functional levels, few studies have used metagenomics to study the effects of starters on rumen development in lambs, especially the effects of feeding with diets containing different NDF/starch ratios on the rumen microbiome of lambs. Metagenomics is a powerful new tool for understanding the composition and function of complex microbial communities, and it allows the abundance of all genes present in the microbial community to be determined and metabolic pathways to be predicted (13). In this paper, we applied metagenomics to analyze the effects of starters with different NDF/starch ratios on the growth, development and rumen microbes of lambs.

## Materials and methods

### Animals and feeding management

Experimental animals were provided by Lanzhou Tianxin Breeding Co., Ltd., and the experimental animals were raised by Defu Agricultural Technology Co., Ltd., Minqin County, Gansu Province. Feeding and management were conducted as follows: we adopted a single-cage feeding mode and immunized all lambs in strict accordance with the normal management procedures of the feeding company. Before experiments were conducted, the farm and all available instruments were sterilized with sanitizine and alcohol.

### Experimental design

Thirty male lambs of Hu sheep, with a birth weight of 3.0 ± 0.5 kg, were randomly divided into two groups, with 15 lambs in each group. The NDF/starch ratio of the starter was 0.5 (group A) or 1.0 (group C). A total of 100 g of starter was weighed out in the morning of the 1st day, and then the appropriate feeding amount for the day was weighed out according to the amount remaining from the previous day every morning (ensuring that the lamb received enough starter). Starting at 10 days of age, each group was fed the same milk replacer (Beijing Precision Animal Nutrition Research Center; nutrition levels: 96.91% dry matter (DM), 23.22% crude protein (CP), and 13.20% ether extract; patent number: ZL02128844.5) and starters with different NDF/starch ratios every day. The milk replacer was fed until 35 days of age, and the starter was fed to the lambs until 56 days of age. During the feeding period, all lambs were free access to drinking water, and the amount of milk replacer was 1.5% of the weight of the lambs as a reference. The starter used in this experiment was produced by Gansu Runmu Biological Engineering Co., Ltd., and was formulated according to the Feeding Standard for Sheep and Goats for Meat (NY/T816-2004). The composition and nutritional composition of the starter are shown in Table 1.

**Table 1:** Composition and nutrient levels of starter (air-dry basis) %.

**Items**	**Starter**	
	**A**	**B**
**Ingredients**		
Alfalfa (%)	11.5	27
Mixed energy feed	57	43
Mixed protein feed (%)	25.5	24.7
Malt root (%)	3.5	3.5
Limestone (%)	1.2	0.5
Premix^a^ (%)	1	1
NaCl (%)	0.3	0.3
Total (%)	100	100
**Nutrient levels** ^b^		
DM (%)	89.9	90.82
CP (%)	21.07	19.89
DE (MJ/kg)	13.58	13.05
Energy and CP ratio	0.64	0.66
Starch (%)	31.66	23.13
NDF (%)	15.81	22.19
NDF/starch	0.5	1
Ca (%)	0.88	0.84
P (%)	0.33	0.35

a The premix provided the following per kg of diets: S 200 mg, Fe 25 mg, Zn 40 mg, Cu 8 mg, Mn 40 mg, I 0.3 mg, Se 0.2 mg, Co 0.1 mg, VB12 0.02 mg, VA 940 IU, VD 111 IU, and VE 20 IU.

b DM, CP, Ca, P, NDF, and starch were measured, while DE was calculated according to the composition and nutrient values of feed in China (2010, 21st ed).

### Sample collection

During the feeding process, the feed amount remaining from the previous day and the feed amount for the day were measured every morning. Lambs were weighed before the start of the experiment, every 7 days thereafter, and again at the end of the experiment before slaughter. After lambs were fed to 56 days of age, six lambs with similar body weights in each group were slaughtered, and the rumen pH was measured by a pH meter immediately after slaughter. Rumen ventral sac tissue samples that were 1 cm^2^ in size were collected after rinsing with normal saline and fixed in 10–15 volumes of 10% neutral formaldehyde solution for tissue sectioning. Moreover, the rumen contents and rumen ventral sac tissues were collected, placed in cryo-storage tubes and immediately placed in liquid nitrogen for temporary storage. After returning to the laboratory, the samples were transferred to a −80°C freezer for storage and subsequent experimental operations. Since there was no significant difference in the macro phenotype production data between the two groups, the rumen contents of the two groups were extracted by metagenomic sequencing for microscopic analysis.

### Sample processing

#### Determination of NH_3_-N and VFA contents and MCP

The NH_3_-N concentration was determined by the phenol-sodium hypochlorite colorimetric method according to Wang et al. (14). The VFA content in rumen fluid was determined by gas chromatography (GC-2010 Plus; Shimadzu, Kyoto, Japan). An internal standard method was used, and the internal standard-based was 2-ethylbutyric acid (2EB). The chromatographic column was an AT-FFAP capillary column (30 m × 0.32 mm × 0.25 μm). The temperature program of the chromatographic column was as follows: 60°C for 1 min, followed by increasing at 5°C/min to 115°C, then at 15°C/min to 180°C. The temperature of the detector was 260°C, and the temperature of the injector was 250°C. MCP concentrations were measured using a kit (Nanjing Jiancheng Institute of Biological Engineering) with a microplate reader (Thermo Fisher Scientific).

#### Calculation of ADFI, ADG, and FCR

ADG (kg/d) = [Weight at the end of the experiment (56 d)-Weight at the beginning of the experiment (10 d)]/experiment period (56 d-10 d);

ADFI (kg/d) = total feed intake during the experiment/experiment period; FCR = ADFI (kg/d)/ADG (kg/d).

#### Preparation and observation of tissue sections

The rumen ventral sac tissue fixed with formaldehyde solution was dehydrated, trimmed, embedded, sliced, stained and sealed. The specific operation procedure was as follows: the fixed tissue was dehydrated by an automatic dehydrator (dehydration duration: 75% alcohol, 4 h; 85% alcohol, 2 h; 95% alcohol, 1 h; 100% alcohol, 0.5 h; 100% alcohol, 0.5 h; 100% alcohol, 0.5 h; 100% alcohol, 0.5 h; 100% alcohol, 0.5 h; 100% alcohol, 0.5 h; xylene, 10 min; xylene, 10 min; paraffin, 1 h; paraffin, 2 h; paraffin, 3 h), embedded, and sectioned. Then, the following operations were conducted: dewaxing with xylene, hematoxylin staining for 10–20 min, rinsing with running water for 1–3 min, differentiation (1% hydrochloric acid and 99% anhydrous ethanol) for 5–10 s, rinsing with running water for 1–3 min, placing into warm (50°C) water or weakly alkaline aqueous solution to turn the solution blue, and rinsing with running water for 1–3 min. Then 85% alcohol was added for 3–5 min, eosin staining was conducted for 3–5 min, and then the samples were rinsed with running water for 3–5 s before alcohol dehydration. Then, the samples were subjected to xylene dewaxing till transparent and transferred to a neutral gum sealing sheet. The slices were dried and photographed with a light microscope (Nikon, Japan). Nipple length, nipple width and base thickness were then measured using Image-Pro Plus software (Media Cybernetics, Bethesda, MD, USA).

### Extraction and detection of total DNA and metagenomic sequencing of rumen contents

The total DNA was extracted by CTAB method after grinding the samples with tissue grinder (Jingxin, Shanghai). Total DNA of the rumen contents was extracted by the CTAB method as follows: (1) a total of 1,000 μl CTAB lysate was added to a 2.0 ml EP tube, 20 μl of lysozyme was added, an appropriate amount of sample was added into the lysate, and the sample was placed in a 65°C water bath (for rumen content samples, the time in the water bath was 2 h). The mixture was homogenized several times during this period to achieve complete lysis. (2) A total of 950 μl of the supernatant was centrifuged, and the same volume of 25:24:1 phenol (pH = 8.0): chloroform:isopentyl alcohol was added. The sample was mixed by inversion and centrifuged at 12,000 rpm for 10 min. (3) The supernatant was collected, isoamyl alcohol (24:1) was added, and the mixture was mixed by inversion and then centrifuged at 12,000 rpm for 10 min. (4) The supernatant was transferred to a 1.5 ml centrifuge tube, 3/4 the volume of isopropyl alcohol was added to the supernatant, and then the mixture was shaken and precipitated at −20°C. (5) The mixture was centrifuged at 12,000 rpm for 10 min, the liquid was removed while taking care to retain the precipitate, the precipitate was washed twice with 1 ml of 75% ethanol, and the remaining small amount of liquid was collected by centrifugation again and removed with a pipette. (6) The sample was blown dry on an ultraclean table or air dried at room temperature (DNA samples were not completely dried to aid in resuspension). (7) A total of 51 μl of ddH_2_O was added to dissolve the DNA samples, which were incubated at 55–60°C for 10 min for dissolution if necessary. (8) A total of 1 μl of RNase A was added to digest RNA and placed at 37°C for 15 min. The purity and integrity of DNA were analyzed by agarose gel electrophoresis (AGE). A Qubit was used to accurately quantify the DNA concentration. For qualified DNA samples, a Covaris ultrasonic fragmentation instrument was used to randomly interrupt fragments with a growth degree of 350 bp, and the whole library was prepared by terminal repair, adding an A tail and sequencing adaptors, and subjected to purification and PCR amplification. Illumina PE150 sequencing was performed by pooling different libraries according to effective concentration and target data volume.

### Bioinformatic analyzes

Based on raw data returned by the Illumina PE150 sequencing platform, data quality control was first carried out: removal of reads with low-quality bases (mass value ≤38) exceeding a certain percentage (default: 40 bp), removal of a certain proportion of reads containing N bases (the default was 10 bp), and removal of reads with overlaps with the adaptor exceeding a certain threshold (the default was 15 bp) were conducted. If the sample was contaminated by the host, it needed to be compared with the host sequence, and the filtered samples could come from the host (15, 16). After pretreatment, clean data were obtained. Second, SOAPdenovo was utilized (17). Assembly software was used for assembly analysis. The assembled scaffolds were interrupted from the N connection to obtain the n-free sequences known as scaftigs (18) (i.e., continuous sequences within Scaffolds). Bowtie 2 software was used to compare clean data of each sample after quality control to scaftigs of each sample after assembly to obtain unused PE reads. Reduced properties were obtained with the following parameters (15, 16): –end-to-end, –sensitive, -I 200, -x 400; unutilized reads of each sample were combined, and k-MER = 55 was selected for mixed assembly (15, 19, 20). Other assembly parameters were the same as those of a single sample. The mixed assembly Scaffolds were interrupted from the N connector to obtain the scaftigs sequences without N. For scaftigs generated from single samples and mixed assemblies, fragments smaller than 500 bp (15, 18, 21, 22) were filtered out, and statistical analysis and subsequent gene prediction were performed.

Starting from a single sample and scaftigs after mixed assembly, MetaGeneMark was used for gene prediction, and genes generated by each sample and mixed assembly prediction were combined to remove redundancy and construct a gene catalog. Starting from the gene catalog (16, 22, 23) and integrating the clean data of each sample, the abundance information of the gene catalog in each sample could be obtained for basic information statistics and core-PAN gene analysis. Species annotation was conducted as follows: DIAMOND software was used to compare genes with various functional databases, and then the LCA algorithm was used to obtain species annotation information (15, 24). Commonly used functional databases were compared, and based on the gene catalog, functional annotation and abundance analysis of metabolic pathways (KEGG), homologous gene clusters (eggNOG) and carbohydrate enzymes (CAZy) were carried out (15, 25–27). Based on the species abundance and functional abundance tables, abundance clustering analysis, NMDS dimensionality reduction analyses, Metastat and linear discriminant analysis effect size (LEfSe) multivariate statistical analyses and metabolic pathway comparative analysis were conducted to mine the differences in species composition and functional composition among samples.

### Data analysis

SPSS software (SPSS Version 25.0, SPSS, Inc.) was used for variance analysis of rumen fermentation parameters and rumen papilla development data; was *P* < 0.05 is considered significant, and 0.05 < *P* < 0.1 was considered a significant trend of difference.

## Results and analysis

### Determination of rumen fermentation parameters

As shown in Table 2, the ADFI, ADG, and MCP in group A was significantly higher than those in group C (*P* < 0.05); the FCR, NH_3_-N, and pH showed no significant difference between groups A and C (*P* > 0.05); the papilla width and muscular thickness of the rumen in group A were significantly higher than those in group C (*P* < 0.05), and the papilla length in group C was higher than that in group A but not significantly (*P* > 0.05). The acetic acid percentage in group A was significantly higher than that in group C (*P* < 0.05), impacting the ratio of acetic acid to propionic acid (A/P).

**Table 2:** Effects of different NDF/starch starter on production performance, rumen fermentation parameters, and rumen papilla development in lambs.

**Items**	**Group**		***p*** **-value**
	**A**	**C**	
NH_3_-N/(mg·dL^−1^)	6.95 ± 1.66	11.79 ± 1.67	0.082
pH	5.92 ± 0.07	6.07 ± 0.03	0.066
MCP (mg·ml^−1^)	7.74 ± 0.59	4.51 ± 0.20	0.002
ADFI (kg·d^−1^)	0.26 ± 0.02	0.21 ± 0.01	0.045
ADG (kg·d^−1^)	0.17 ± 0.01	0.14 ± 0.01	0.022
FCR	1.63 ± 0.07	1.51 ± 0.06	0.236
**VFA**			
Acetic acid, %	67.38 ± 1.98	60.22 ± 1.39	0.015
Propionic acid, %	19.98 ± 1.28	21.77 ± 1.40	0.446
Isobutyrc acid, %	0.70 ± 0.08	0.78 ± 0.08	0.551
Butyric acid, %	10.91 ± 1.01	10.14 ± 1.22	0.639
Isovaleric acid, %	0.74 ± 0.07	0.72 ± 0.09	0.851
Valerianic acid, %	2.59 ± 0.28	3.22 ± 0.22	0.132
A/P	3.63 ± 0.36	2.72 ± 0.22	0.058
TVFA, mmol/L	73.82 ± 10.19	63.05 ± 6.96	0.392
**Rumen papillae**			
Papillae length, μm	1,179.76 ± 67.89	1,381.46 ± 52.01	0.053
Papillae width, μm	440.17 ± 17.72	365.30 ± 10.11	0.001
Tunica muscularis thickness, μm	1,188.07 ± 58.77	968.92 ± 38.45	0.005

MCP, Microbial protein; ADFI, average daily feed intake; ADG, average daily gain; FCR, feed conversion ratio; TVFA, Total volatile fatty acids; A/P, the ratio of acetic acid to propionic acid.

The unit % represents the proportion of a single VFA in the TVFA.

### Gene prediction results and abundance analysis

Open reading frame (ORF) prediction of scaftigs (≥500 bp) was performed using MetaGeneMark and the prediction results and basic information of the gene catalog obtained are shown in Table 3. A total of 1,315,345 ORFs were predicted, with an average of 109,612 ORFs per sample. After redundancy removal, a total of 407,675 genes in the gene catalog were obtained for subsequent species annotation and functional analysis. The total length of the non-redundant gene catalog was 331.68 Mbp, the average length was 813.58 bp and the GC content was 47.93%.

**Table 3:** Gene catalog.

**Sample name**	**ORFs NO**	**Integrity: all**	**Total length (Mbp)**	**Average length (bp)**	**GC (%)**
A13	87,584	55,495 (63.36%)	69.93	798.41	50.80
A14	76,546	41,431 (54.13%)	56.65	740.14	51.70
A16	90,072	62,937 (69.87%)	74.33	825.20	50.31
A17	89,586	54,626 (60.98%)	69.80	779.15	47.58
A18	97,211	57,291 (58.93%)	74.64	767.85	51.25
A19	96,432	56,624 (58.72%)	73.78	765.07	48.56
C13	116,682	68,065 (58.33%)	88.75	760.65	48.91
C14	141,464	89,441 (63.23%)	113.15	799.85	48.47
C15	114,493	73,339 (64.06%)	90.68	792.03	49.40
C16	140,327	84,905 (60.51%)	109.49	780.25	48.31
C19	138,608	95,946 (69.22%)	115.24	831.39	48.17
C20	126,340	82,830 (65.56%)	102.58	811.97	48.70

ORFs NO. represents the number of genes in the gene catalog; Integrity: all represents the percentage of the number of complete genes (both start and stop codons); Total Len. (Mbp) represents the total length of genes in the gene catalog, in millions; Average Len. represents the average length of genes in the gene catalog; GC percent represents the overall GC content of genes in the predicted gene catalog.

**Figure 1:**
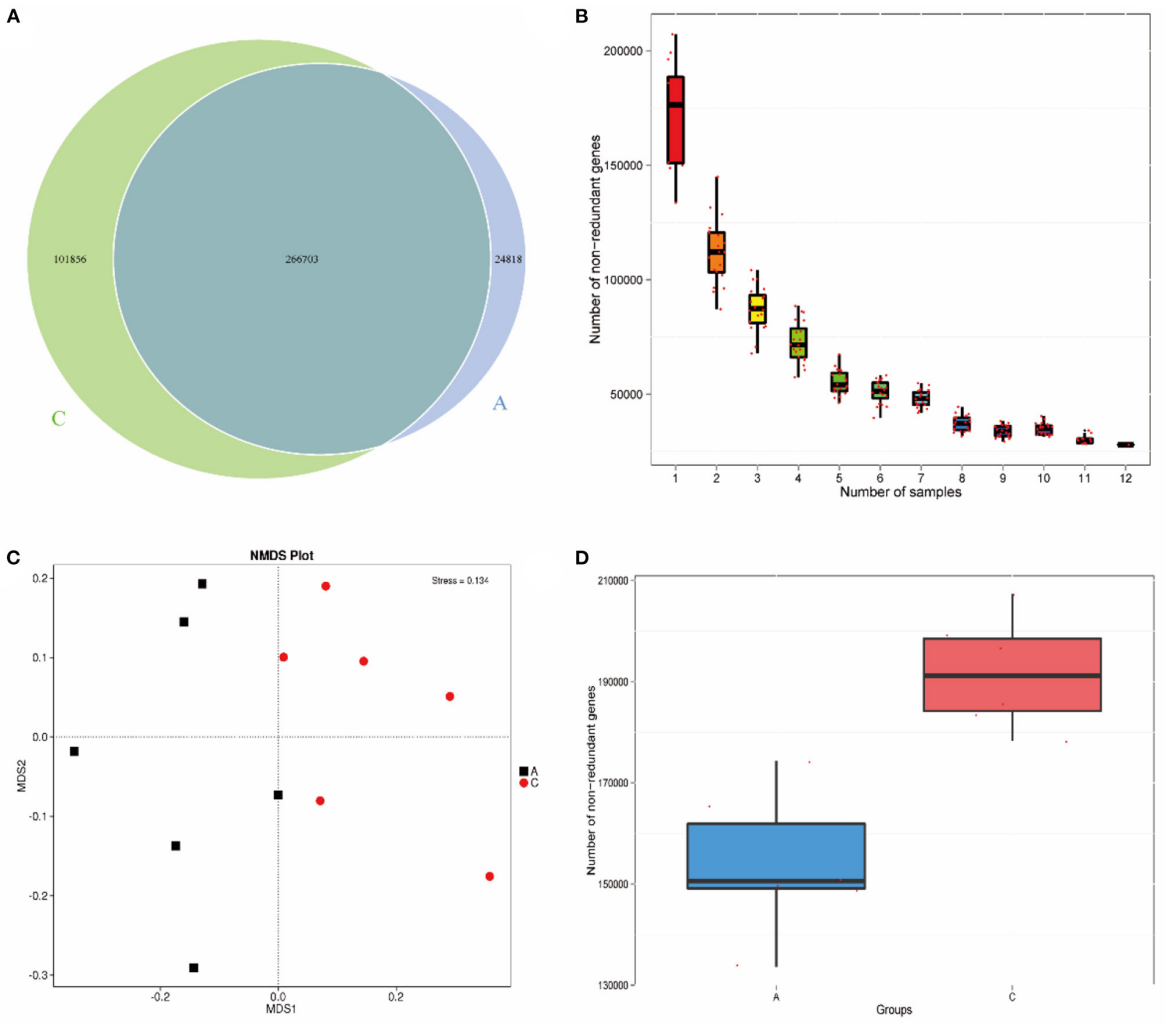
Venn diagram of number of genes **(A)**; Core gene dilution curve **(B)**; NMDS plot based on phylum level **(C)**; Box plot of gene number difference between groups **(D)**.

A Venn diagram (Figure 1A) showed 266,703 common genes between the two groups, 24,818 unique genes in group A and 101,856 unique genes in group C. Based on the abundance information of each gene in the gene catalog of each sample, core gene analysis (Figure 1B) was performed. As the number of samples increased, the number of genes in sample combinations gradually stabilized, and additional samples only increased the number of genes in a small number of sample combinations, indicating that the sequencing was reasonable and could meet the requirements of subsequent analysis. The NMDS plot (Figure 1C) shows that the two groups of samples are located on both sides of the horizontal axis, and the distance between the samples in each group is close, indicating that there is a significant difference between the two groups. To investigate the different gene numbers between groups, a box diagram of gene number difference between groups was drawn, and the results are shown in Figure 1D.

### Species annotation and differential analysis

**Figure 2:**
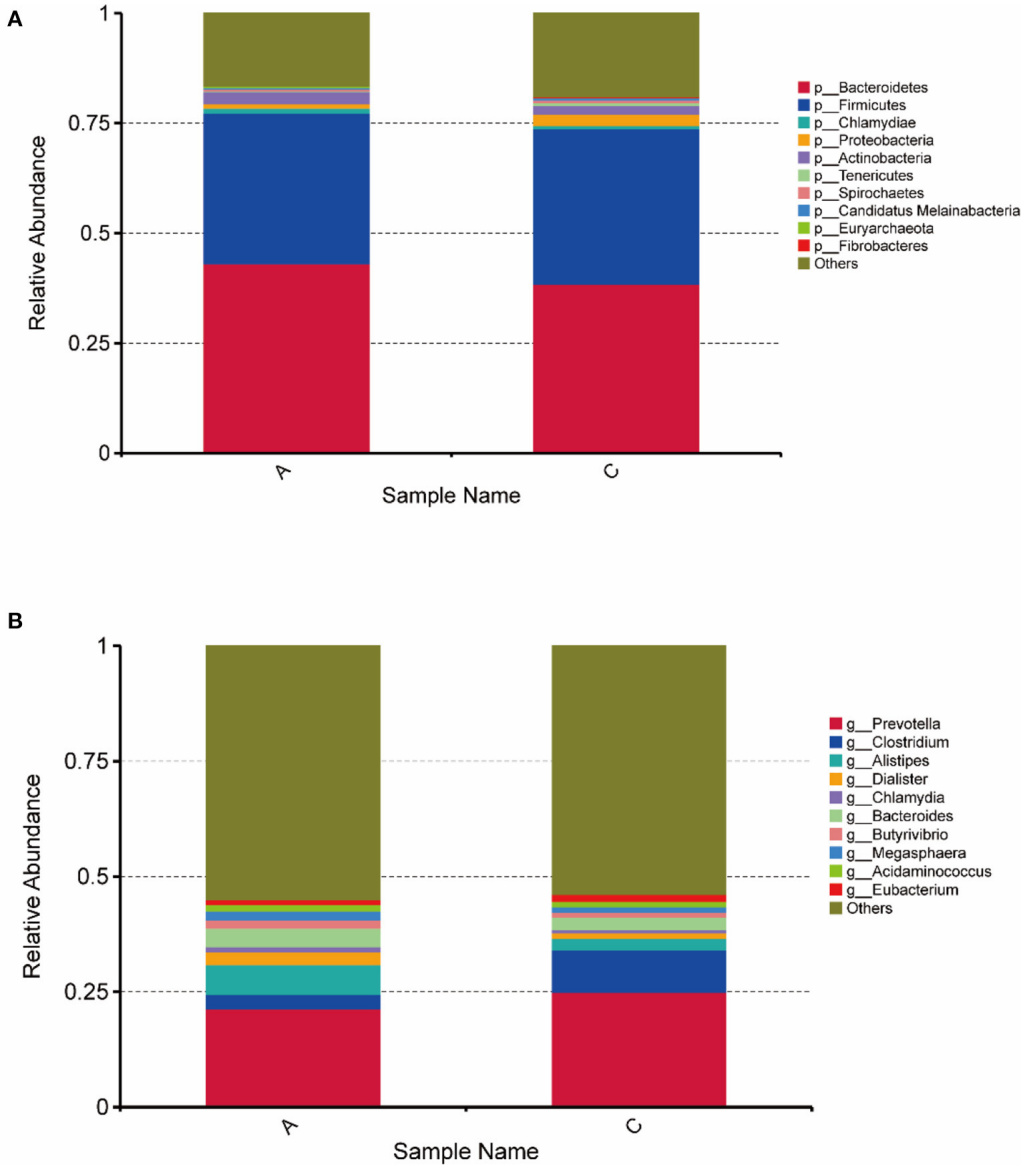
Histogram of horizontal relative abundance of phylum **(A)** and genus **(B)**. The horizontal axis represents the sample name; the vertical axis represents the relative proportion of species annotated to a particular type; the legend on the right shows the species category corresponding to each color block.

In this study, DIAMOND software was used to compare unigenes with each functional database. For the comparison results of each sequence, the results with the highest score were selected for subsequent analysis, and the relative abundance of different functional levels was calculated. The unigenes for each sample (407,675) were compared with the NR database, and the number of genes that could be annotated in the NR database was 343,192 (84.18%). In the NR database that could be annotations, the proportions of genes annotated at the bacterial kingdom, phylum, class, order, family, genus and species levels were 90.07, 86.29, 82.88, 82.40, 74.59, 69.91, and 47.75%, respectively. For the top 10 species in terms of relative abundance at the phylum and genus levels, a histogram of relative abundance was generated (Figure 2). Figure 2 shows that Bacteroidetes (43.07%), Firmicutes (34.25%), Actinobacteria (2.82%) and Chlamydiae (1.17%) were the main Bacteroides in group A. Bacteroidetes (38.46%), Firmicutes (35.32%), Proteobacteria (2.58%), and Actinobacteria (1.98%) were the main Bacteroidetes in group C. Prevotella (21.34%), Alistipes (6.42%,) Bacteroides (3.98%), Clostridium (3.16%), and Dialister (2.76%) were the main genera in group A. Prevotella (24.99%), Clostridium (9.14%), Bacteroides (2.68%), and Alistipes (6.42%) were the main genera in group C. These were the top 10 bacteria in relative abundance at the genus level, among which the abundance of seven species in group A was higher than that in group C, and the abundance of the other three species in Group C was higher than that in group A. The abundance of Alistipes and Bacteroides in group A was significantly higher than that in group C (*P* < 0.05); the abundance of Clostridium was significantly higher in group C than in group A (*P* < 0.05).

### LEfSe analysis of differentially abundant species between groups

**Figure 3:**
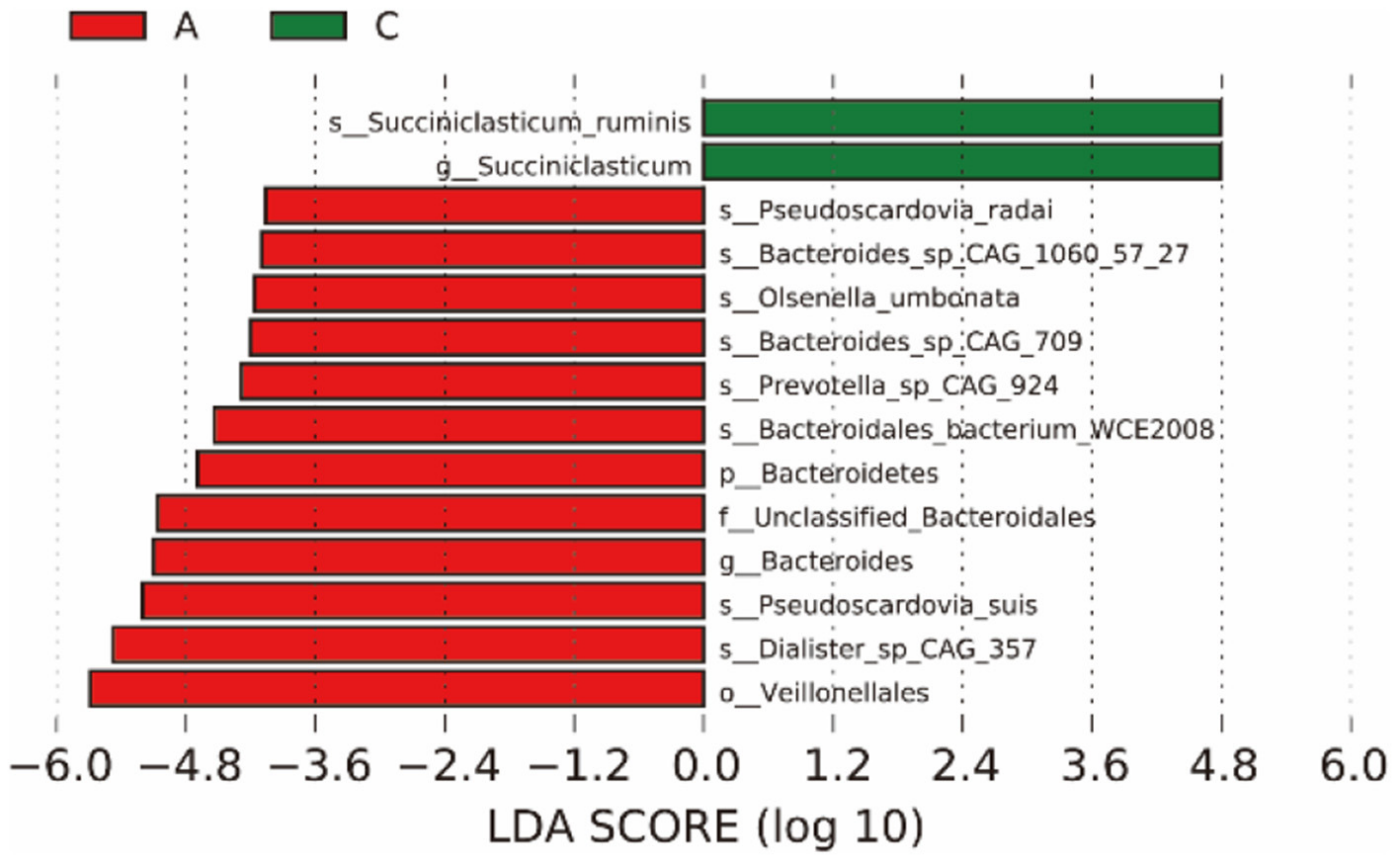
The left figure shows the LDA value distribution of different species. The histogram of LDA value distribution shows the species whose LDA score is greater than the set value (the default value is 4), that is, the Biomarker with statistical difference between groups. The length of the bar graph represents the impact of different species (LDA score).

LEfSe analysis (Figure 3) showed that the biomarker of group A and group C were significantly different (LDA score > 4), there were 14 differentially abundant biomarkers in group A, and there were significant differences between groups A and C.

### Metastat analysis of intergroup differentially abundant species

**Figure 4:**
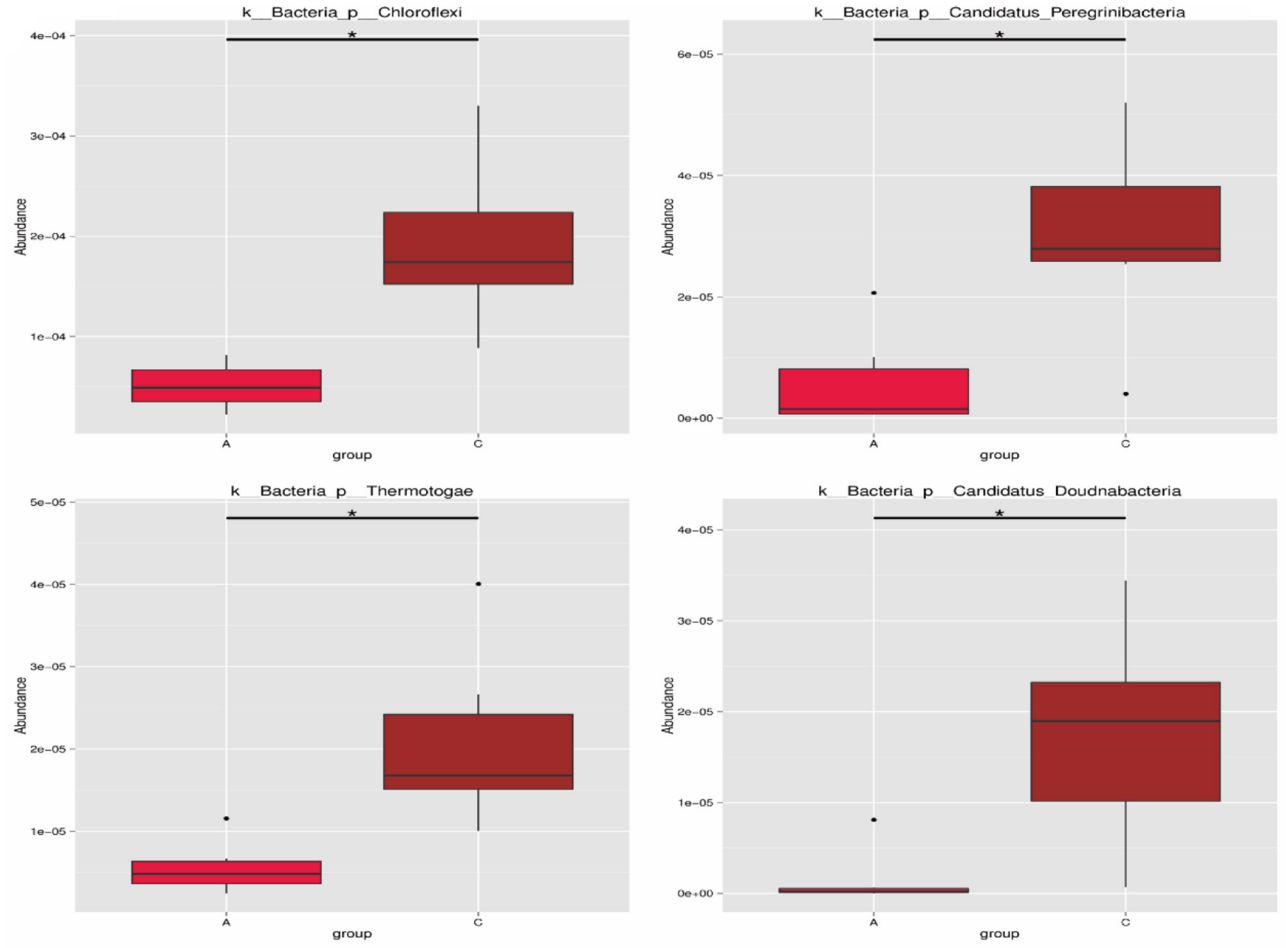
Phylum level differential species. The horizontal axis is the sample grouping, and the vertical axis is the relative abundance of corresponding species; the horizontal lines represent two groups with significant differences. ^*^Represents significant difference between the two groups (*q* < 0.05), ^**^Represents extremely significant difference between the two groups (*q* < 0.01).

As shown in Figure 4, there were four phyla with significant differences between group A and group C at the phylum level. The relative abundances of Chloroflexi, Candidatus Peregrinibacteria, Thermotogae, and Candidatus Doudnabacteria in group C were both significantly higher than those in group A (*P* < 0.05).

### KEGG database functional annotation and abundance analysis

**Figure 5:**
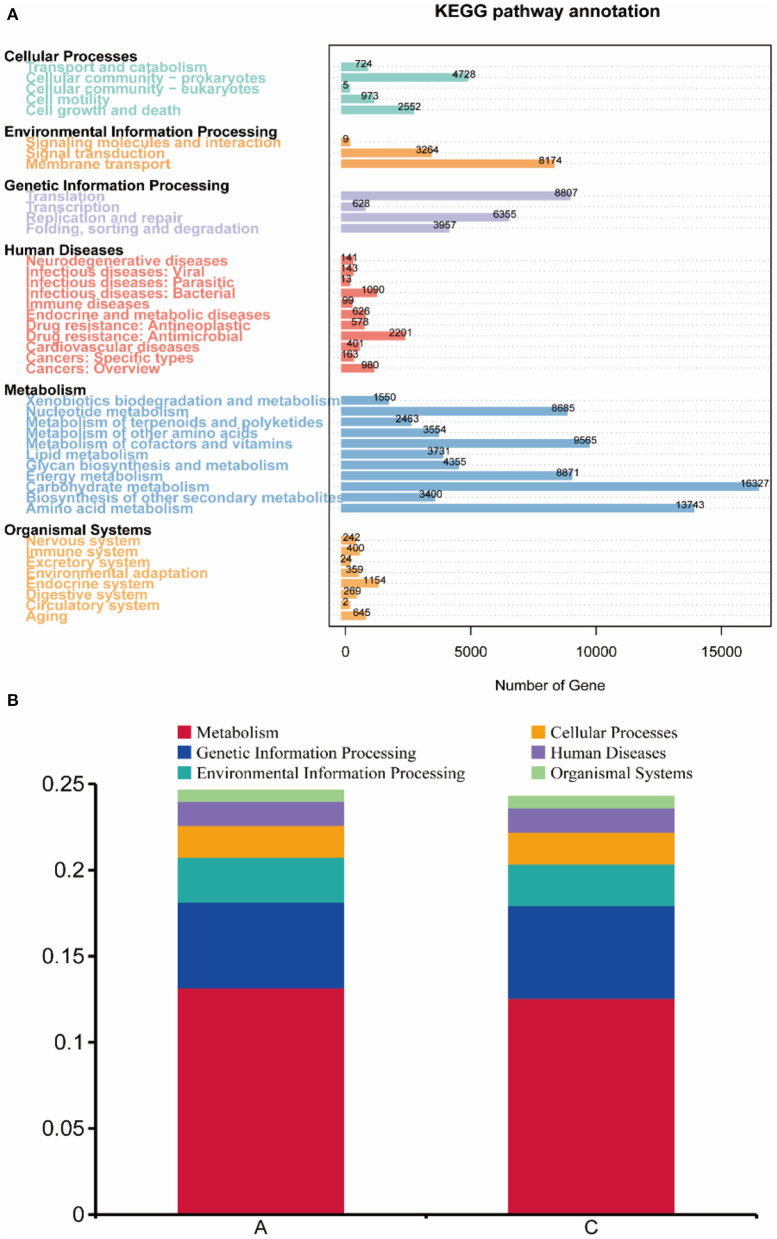
**(A)** Statistical map of gene number. The number of genes annotated into the KEGG metabolic pathways in rumen microorganisms of the two groups was calculated. **(B)** Functional annotation abundance graph (level 1).

Unigenes (407,675) of each sample were matched with the KEGG database, 261,718 (64.20%) genes were matched with the KEGG database, and 141,546 (34.72%) genes were matched with 3,891 Kos (KEGG ortholog groups). The statistical diagram of the number of genes annotated into KEGG metabolism pathways in rumen microbes of lambs in group A and group C is shown in Figure 5. As shown in the figure, metabolism-related genes accounted for a large proportion of the genes, among which carbohydrate metabolism was the most abundant functional category, and 16,327 genes were annotated in this category. The abundance of genes involved in amino acid metabolism was high, with 13,743 genes being annotated in this category. Nucleotide metabolism, metabolism of cofactors and vitamins and energy metabolism were also abundant. There were 8,982 genes annotated for cellular processes, 11,447 genes for environmental information processing, 19,747 genes for genetic information processing, 6,435 genes for human diseases, and 3,095 genes for organismal systems. In addition, pathway with the highest gene abundance in environmental information processing was membrane transport, which had 8,174 genes. In genetic information processing, translation had the highest gene abundance, with 8,807 genes.

After analyzing the abundance of each function at level 1 (Figure 5), in group A metabolism accounted for 13.16% of gene function, genetic information processing accounted for 4.99%, environmental information processing accounted for 2.60%, cellular processes accounted for 1.84%, human diseases accounted for 1.40%, and organismal systems accounted for 0.63%. In group C metabolism accounted for 12.56% of gene function, genetic information processing accounted for 5.40%, environmental information processing accounted for 2.41%, cellular processes accounted for 1.83%, human diseases accounted for 1.43%, and organismal systems accounted for 0.64%. These were not statistically significant differences in abundance (*P* < 0.05).

### Functional annotation and abundance analysis based on the eggNOG database

**Figure 6:**
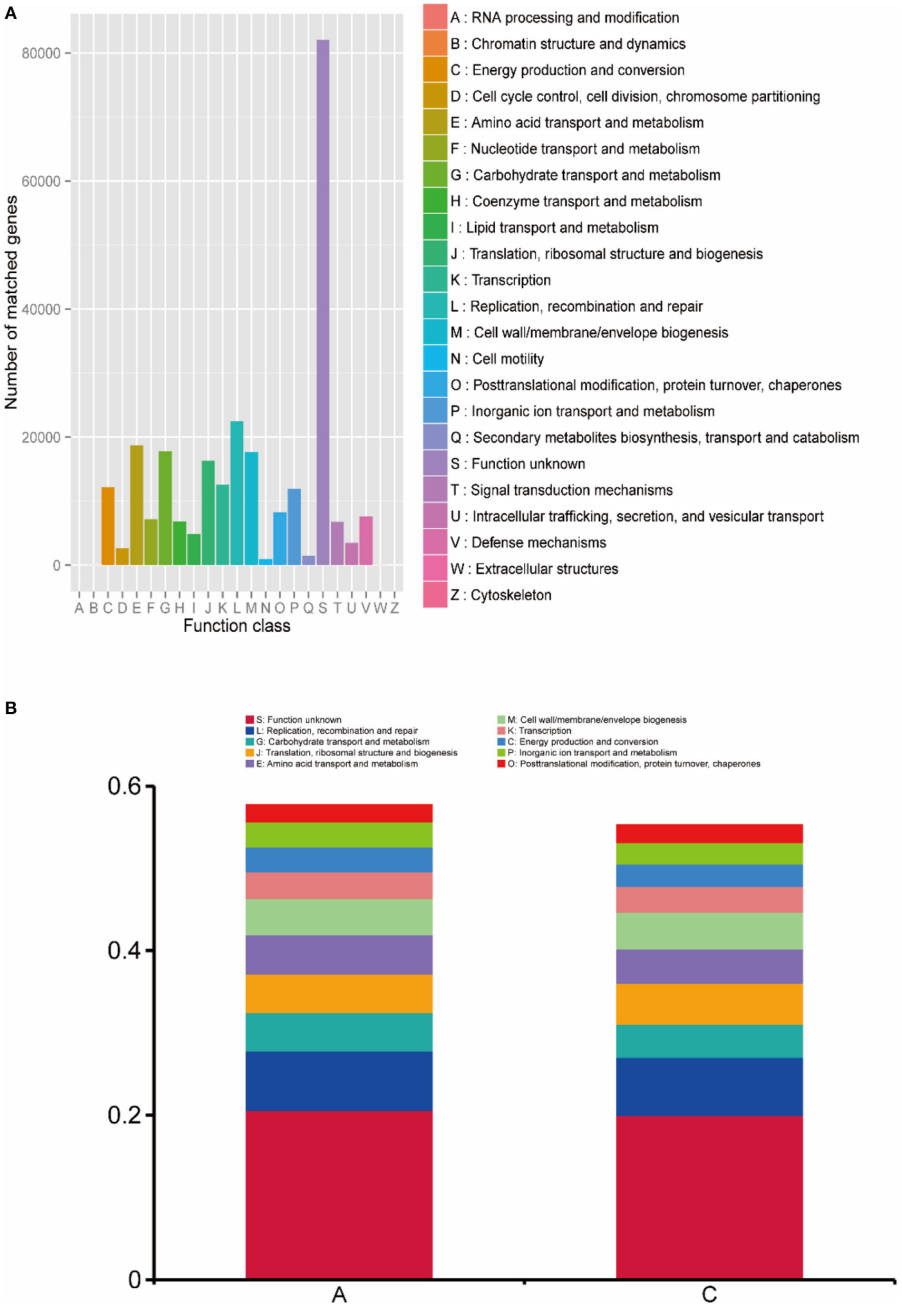
**(A)** Statistics of the number of gene annotations based on the eggNOG database; **(B)** the histogram of functional abundance on level 1 based on the KEGG database.

The functional annotation results of the eggNOG database are shown in Figure 6A, and the main functional genes included 12,173 genes for energy production and conversion; 18,668 genes for amino acid transport and metabolism; 17,768 genes for carbohydrate transport and metabolism; 16,301 genes for translation, ribosomal structure and biogenesis; 12,592 genes for transcription; 22,471 genes for replication, recombination and repair; 17,671 genes for cell wall/membrane/envelope biogenesis; and 11,935 genes for inorganic ion transport and metabolism. In addition, unknown functional genes accounted for a large proportion of the total genes.

The functional abundance of lamb intestinal microbes was analyzed at level 1, and the analysis results are shown in Figure 6B. In group A, genes of unknown function accounted for 20.58% of gene function; replication, recombination and repair accounted for 7.25%; carbohydrate transport and metabolism accounted for 4.66%; translation, ribosomal structure and biogenesis accounted for 4.73%; amino acid transport and metabolism accounted for 4.71%; cell wall/membrane/envelope biogenesis accounted for 4.44%; transcription accounted for 3.24%; energy production and conversion accounted for 3.01%; inorganic ion transport and metabolism accounted for 3.03%; and posttranslational modification, protein turnover, chaperones accounted for 2.07%. For group C, genes of unknown function accounted for 19.99% of gene function; replication, recombination and repair accounted for 7.08%, carbohydrate transport and metabolism accounted for 4.00%, translation, ribosomal structure and biogenesis accounted for 5.01%; amino acid transport and metabolism accounted for 4.15%; cell wall/membrane/envelope biogenesis accounted for 4.44%; transcription accounted for 3.15%; energy production and conversion accounted for 3.01%; inorganic ion transport and metabolism accounted for 2.74%; and posttranslational modification, protein turnover, chaperones accounted for 2.15%. There was no significant difference between the two groups (*P* > 0.05).

### CAZy database functional annotation and abundance analysis

**Figure 7:**
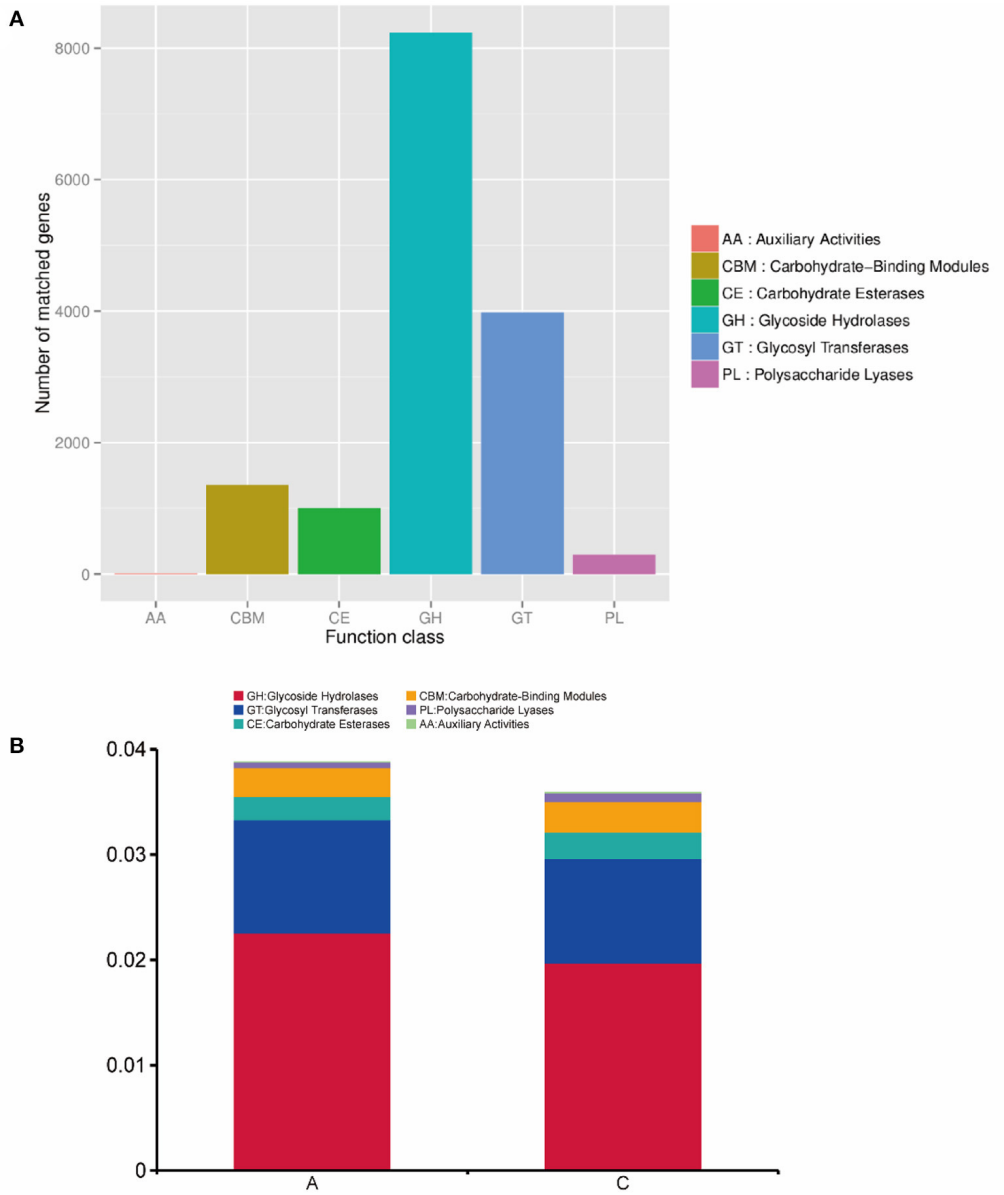
**(A)** Statistics of the number of gene annotations based on the CAZy database; **(B)** the histogram of functional abundance on level 1 based on the CAZy database.

We annotated the carbohydrate enzymes contained in groups A and C, and the annotation results are shown in Figure 7A. Seven genes were attributed to auxiliary activities (AAs), 1,355 genes to carbohydrate-binding modules (CBMs), 1,000 genes to carbohydrate esterases (CEs), 8,237 genes to glycoside hydrolases (GHs), 3,980 genes to glycosyl transferases (GTs), and 293 genes to polysaccharide lyases (PLs). The functional abundance of rumen microorganisms in lambs was analyzed at level 1, and the analysis results are shown in Figure 7B.

In group A, AA genes accounted for 0.0003% of the abundance, CBM genes accounted for 0.27%, CE genes accounted for 0.22%, GH genes accounted for 2.25%, GT genes accounted for 1.08%, and PL genes accounted for 0.05%. In group C, AA genes accounted for 0.0026% of the abundance, CBM genes accounted for 0.29%, CE genes accounted for 0.25%, GH genes accounted for 1.97%, GT genes accounted for 0.99%, and PL genes accounted for 0.08%. AA genes in group C were significantly enriched in group A (*P* < 0.05), while the abundances of other genes were not significant between the two groups (*P* > 0.05).

### Differential function analysis

Based on MetaStats analysis, we screened genes with *P* < 0.05 and |log_2_(FC)| ≥ 1 as significantly different genes. Table 4 shows the statistics of significantly different genes in the two groups. At KEGG level 2, the cell motility of group C was significantly higher than group A (*P* < 0.05). At CAZy level 2, GT39, GT1, CBM58, and CBM66 were significantly higher in group A than in group C (*P* < 0.05), and PL16, CBM77, GT84, CBM61, CBM51, GT39, GH84, PL9, CE8, CBM42, GH119, CBM69, and PL10 were significantly more abundant in group C (*P* < 0.05).

**Table 4:** Different functions of rumen microbiota at level 1 or level 2 based on KEGG and CAZy database.

**Item**	**A**	**C**	**log_2_(FC)**	***P*** **-value**
**KEGG level 2**				
Cellular processes; cell motility	0.07%	0.15%	1.09	0.017
**CAZy level 2**				
PL16	0.00007%	0.00066%	3.27	0.004
CBM77	0.00010%	0.00088%	3.22	0.005
GT84	0.00070%	0.00629%	3.18	0.007
CBM61	0.00023%	0.00135%	2.57	0.008
CBM51	0.00003%	0.00072%	4.54	0.010
GT39	0.00272%	0.00134%	−1.02	0.015
GH84	0.00023%	0.00133%	2.53	0.015
PL9	0.00053%	0.00191%	1.85	0.024
GT1	0.01281%	0.02644%	1.05	0.024
CE8	0.01501%	0.03123%	1.06	0.033
CBM42	0.00001%	0.00008%	2.53	0.034
GH119	0.00001%	0.00121%	6.73	0.038
CBM69	0.00001%	0.00149%	6.89	0.039
PL10	0.00422%	0.01259%	1.58	0.042
CBM58	0.00635%	0.00238%	−1.42	0.042
CBM66	0.00681%	0.00293%	−1.22	0.044

FC, fold changes; log2(FC), log base 2 of the fold changes of group A and group C.

MetaStats analysis found that there was no significant difference in metabolic pathways between groups A and C. In the metabolic pathway diagram (Supplementary Figure 1) of starch and sucrose metabolism (map00500), maltose phosphorylase [EC:2.4.1.8] is a unique enzyme to group C and is produced by *Faecalibacterium* sp. CAG:74_58_120.

## Discussion

### Effects of starters with different NDF/starch ratios on the production performance, rumen fermentation characteristics, and rumen tissue morphology of Hu sheep

In this experiment, we found that starter feeding in group A improved the ADG and ADFI of lambs, and there was no difference in FCR between the two groups, but the lambs in group A needed for weight gain, and the lambs in group A needed more concentrate feed, so the starter in group C has more economic value in terms of feed benefits. The pH, NH_3_-N content, and VFA content are important indicators of rumen fermentation in ruminants (28). pH can be affected by many factors, such as high doses of starch (29, 30). Starch and cellulose are the main dietary components of ruminants, and the degradation of starch and cellulose in ruminants is the key to their high yield (31). In this study, the low starch content in Group C led to a high pH value, which was consistent with the results of Liu et al. (28). The NH_3_-N concentration comprehensively reflected the degradation of dietary nitrogen in the rumen and the utilization of ammonia by microorganisms, mainly reflecting the utilization of nitrogen by ruminants (32–34). Contrary to a previous study (28), in this study, high concentrate feed levels did not increase the NH_3_-N concentration, but *Prevotella* degraded peptides in the rumen and therefore may promote the formation of NH_3_-N, which may be the reason for the high NH_3_-N concentration in Group C. Many studies have shown that the acetic acid concentration in diets with high concentrate levels is lower and the propionic acid concentration is significantly increased (28, 35). The results of this study contradict these findings, since animals with high feed efficiency are generally thought to produce more VFAs and less methane (36, 37), while the high acetic acid levels in group A is more likely to cause methane emissions, which is not conducive to feed conversion ratio. At the same time, the decrease in the *A*/*P*-value of group C reflects the improvement in the energy utilization efficiency of group C starter. Rumen development affects nutrient digestion and performance in adulthood, and can be measured from the developmental status (length and width) of the rumen papilla, affecting the digestive capacity and yield of adult lambs (38). In this study, the rumen papilla length in group A was lower than that in group C, indicating that group C starter can promote the growth of rumen papillae, which is consistent with our previous research results showing that high NDF feeding can promote rumen papilla development (33).

### Effects of starters with different NDF/starch ratios on rumen microbial species abundance in Hu lambs

Metagenomic sequencing analysis revealed the complex and diverse microbial population in the rumen. Recent studies have shown that rumen microbial community diversity has a direct impact on ruminant performance and the rumen environment (39, 40). Ruminants rely on rumen microorganisms to degrade roughage, which provides energy and protein nutrients for the maintenance of growth and lactation in host animals (41). The rumen microflora structure of lambs fed starters with different NDF/starch ratios was analyzed to understand the relationship between host and microbial community, which is conducive to screening the starter for early development of lambs. There was no significant difference in the horizontal abundance ofphyla between groups A and C. Four main phyla existed in the rumen, more than 70% of which was accounted for by Bacteroidetes, which was the most abundant, followed by Firmicutes, Actinobacteria, Proteobacteria, and Chlamydia, which is consistent with previous reports (42–44). In this study, Bacteroidetes and Firmicutes had the highest microbial abundance in the rumen of lambs, which was consistent with previous reports (45, 46).

The species annotation showed that the abundance of Bacteroidetes in group A was higher than that in group C. Bacteroidetes can degrade carbohydrates (47) and are mainly responsible for protein hydrolysis, carbohydrate degradation and amino acid fermentation to acetic acid. It is concluded that the NDF/starch ratio of group A as more effective than that of group C in promoting the growth of Bacteroidetes in the rumen of lambs and in degrading carbohydrates in feed. However, the abundance of Firmicutes in group C was higher than that in group A, which played an important role in energy conversion (47, 48). Therefore, the diet in group C could promote the propagation of Firmicutes and increase the energy conversion efficiency. Consistent with the results of this study, *Prevotella* has been reported as the most abundant rumen genus (49), and the abundance of *Prevotella* in group A and group C were 21.34 and 24.99%, respectively. *Prevotella* was positively correlated with VFA concentration, indicating that *Prevotella* plays an important role in VFA biosynthesis (50). Although this has not been fully demonstrated in the microbial profile of feed efficiency, *Prevotella* has been shown to be more abundant in inefficient animals (51). The study also showed that *Prevosella* abundance was related to propionic acid production and positively correlated with NH_3_-N (52) content, which was also consistent with our experimental results. Because *Prevotella* degrades peptides in the rumen, it may promote the formation of NH_3_-N (53).

Among the four genera with significant differences at the phylum level, Chloroflexi is known to be related to methanogenesis (54), but Chloroflexi has a low abundance in the rumen and produces limited methane compared to methanogens, which can produce methane using acetic acid. Members of Candidatus Peregrinibacteria are involved in carbohydrate metabolism and amino acid synthesis (55), which may be because high NDF starter promoted the mass propagation of microorganisms involved in carbohydrate metabolism. Thermotogae members can grow on simple and complex carbohydrates and have a high H_2_ production capacity, thereby reducing greenhouse gas emissions (56), which inhibits Chloroflexi methanogenesis. However, the function of Candidatus Doudnabacteria is currently unclear.

### Functional research based on different databases

We used comparisons of different databases to predict the function of the lamb rumen microbiome. The results showed that the rumen microbial genes of lambs in the two groups were mainly involved in carbohydrate metabolism and amino acid metabolism, and there was no significant difference in the metabolic process between the two groups, which may be because in addition to the feeding environment, the type and source of starter were also important influencing factors. In addition, compared with previous studies (57, 58), the current study indicate that the overall function of the rumen bacterial community is mainly related to membrane transport, carbohydrate metabolism, amino acid metabolism, replication and repair, translation and energy metabolism. Our data also showed that the difference in rumen bacterial community function between the two groups mainly occurred in metabolism. Specifically, compared with group A, there were more genes related to genetic information processing in group C, and the abundance of genes related to replication and repair was significantly higher than that in group A, while genes related to metabolism and environmental information processing were enriched in group A. Therefore, we speculated that some bacteria in group C regulate their own growth and cell differentiation through the growth and apoptosis of endocrine hormone cells to meet the needs of animals for rich nutrient metabolism and that rumen bacteria in group A can help lambs accumulate more energy.

Carbohydrates are important organic compounds and the main energy source for living cells. Carbohydrates in the gastrointestinal tract play an important role in providing nutrition for hosts and microorganisms or regulating the complex relationship between them (59). Carbohydrate-active enzymes can decompose macromolecular carbohydrates, and various carbohydrate-active enzymes cooperate with each other to degrade oligosaccharides and polysaccharides. The more complex the polysaccharide is, the greater the number of carbohydrate-active enzymes required (60). Studies have shown that the abundance of the gastrointestinal flora increases with increasing dietary fiber intake (61), and a large amount of dietary fiber cannot be digested and decomposed by lambs themselves, requiring a series of enzymes to synergistically aid in degradation. For example, the main chain of polysaccharides is mainly degraded by GHs and partially degraded PLs, and the side chain is degraded by hydrolases such as xylosidase mannosidase and CEs (62). CBMs themselves do not show enzyme activity but help in the binding of GHs to polysaccharides and enhance their activity (63, 64). CE (65) is responsible for the cleavage of glycosidic bonds, and PLs (66) and AAs (67), which target insoluble polymers, are in the CAZymes class with relatively little annotation in the metagenome studied. PL9 and PL10 are members of the pectinase family (68), members of which catalyze the hydrolysis of A-1, 4-glycosidic bonds (69, 70) and play an important role in the degradation and modification of pectin (71, 72). Glycosyltransferases (GTs), as natural biocatalysts, can catalyze the transfer of glycosylates from activated sugar donors to different receptors (72, 73). The GT1 family is a reversal enzyme (74). GT1 family enzymes are common in most organisms, such as bacteria and animals (75), but members from different fields show different functions. For example, “antibiotic glycosylation” is common in microorganisms (76). The GT1 family plays a crucial role in antibiotic biosynthesis and antibiotic resistance (75). In view of these results, the enrichment of CAZyme genes (GH, CE, PL, AA, and CBM) encoding carbohydrate decomposition in the rumen microbial community of group C demonstrated that the group C lambs had a better ability to degrade complex substrates.

In starch and sucrose metabolism, both groups had multiple metabolic pathways and 56 enzymes. The unique enzyme in group C was maltose phosphorylase [EC:2.4.1.8], which is mainly responsible for the degradation of maltose into glucose (77), and this additional metabolic pathway is beneficial for host decomposition of dietary nutrients.

## Conclusions

In conclusion, in group C, with an NDF/starch ratio of 1.0, the rumen papillae of lambs were well-developed, the energy utilization efficiency was improved, and CAZyme genes were enriched in the rumen microbial community of lambs. Moreover, group C had greater economic value from the perspective of feed benefits.

## Data availability statement

The datasets presented in this study can be found in online repositories. The names of the repository/repositories and accession number(s) can be found in the article/Supplementary material.

## Ethics statement

The animal study was reviewed and approved by the Faculty Animal Policy and Welfare Committee of Gansu Agricultural University.

## Author contributions

HZ was responsible for pilot implementation, sample collection and analysis, manuscript preparation, and manuscript submission and revision. FL was involved in data analysis, statistical analysis, manuscript language revision, journal selection, and manuscript submission and revision. GL participated in the experimental design, experimental implementation, and sample collection. XP performed sample analysis, scanning electron microscopy, and data collection and analysis. XW contributed to supervision, management, assisted students in managing animals, and collecting and analyzing samples. All authors contributed to the article and approved the submitted version.
