# Supplementary figures and images for: Effects of starters with different NDF/starch ratio on rumen fermentation parameters and rumen microorganisms in lambs
Source: Front Vet Sci. 2023 Jan 26;10:1064774. doi: 10.3389/fvets.2023.1064774 (PMC9911143; doi:10.3389/fvets.2023.1064774)

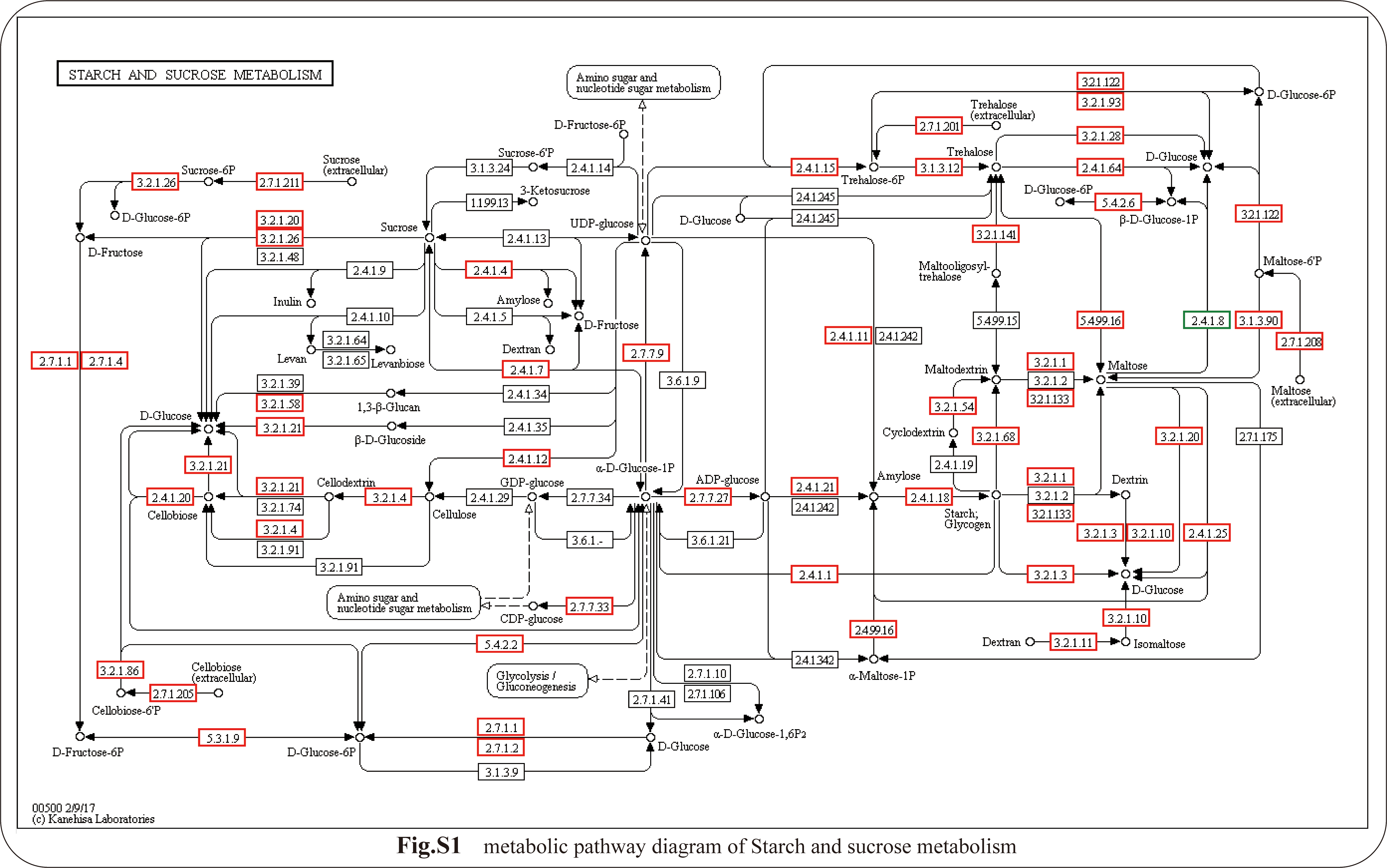

Supplement: Supplementary file 1 [file Image_1.TIF]
